# Antibacterial and Anti-Adherence Efficacy of Silver Nanoparticles Against Endodontic Biofilms: An In Vitro and Ex Vivo Study

**DOI:** 10.3390/pharmaceutics17070831

**Published:** 2025-06-26

**Authors:** Mariana Goretti Pérez-Sáenz, Rita Elizabeth Martínez-Martínez, Erasto Armando Zaragoza-Contreras, Rubén Abraham Domínguez-Pérez, Simón Yobanny Reyes-López, Alejandro Donohue-Cornejo, Juan Carlos Cuevas-González, Karla Lizette Tovar-Carrillo, Erika de Lourdes Silva-Benítez, José Luis Ayala-Herrera, León Francisco Espinosa-Cristóbal

**Affiliations:** 1 Master Program in Dental Sciences, Stomatology Department, Institute of Biomedical Sciences, Autonomous University of Juarez City (UACJ), Envolvente del PRONAF and Estocolmo s/n, Ciudad Juárez 32310, Chihuahua, Mexico; mariana.perez@uacj.mx (M.G.P.-S.); adonohue@uacj.mx (A.D.-C.); juan.cuevas@uacj.mx (J.C.C.-G.); karla.tovar@uacj.mx (K.L.T.-C.); 2 Master Program in Advanced Dentistry, Faculty of Dentistry, Autonomous University of San Luis Potosi, Manuel Nava Avenue, University Campus, San Luis Potosí 78290, San Luis Potosí, Mexico; rita.martinez@uaslp.mx; 3 Department of Engineering and Materials Chemistry, Centro de Investigación en Materiales Avanzados, S. C., Miguel de Cervantes No.120, Chihuahua 31136, Chihuahua, Mexico; armando.zaragoza@cimav.edu.mx; 4 Laboratory of Multidisciplinary Dental Research, Faculty of Medicine, Autonomous University of Queretaro, Clavel Street, Prados de La Capilla, Santiago de Querétaro 76176, Querétaro, Mexico; dominguez.ra@uaq.mx; 5 Institute of Biomedical Sciences, Autonomous University of Juarez City (UACJ), Envolvente del PRONAF and Estocolmo s/n, Ciudad Juárez 32310, Chihuahua, Mexico; simon.reyes@uacj.mx; 6 Master Program in Advance Oral Rehabilitation, Faculty of Dentistry, Autonomous University of Sinaloa, Josefa Ortiz de Domínguez Street, Universitary Campus, Universitaria, Culiacán 80010, Sinaloa, Mexico; erikasilva@uas.edu.mx; 7 Faculty of Dentistry, La Salle Bajío University, Paseo de los Ángeles and Medina Street, León 37000, Guanajuato, Mexico; jayala@lasallebajio.edu.mx

**Keywords:** metallic nanoparticles, silver, anti-infective agents, biofilms, root canal irrigants, *Enterococcus faecalis*

## Abstract

**Background/Objectives:** Root canal infections represent a serious challenge to the success of endodontic treatment. The most commonly used antimicrobial irrigants, such as sodium hypochlorite (NaOCl), have certain limitations, while endodontic biofilms pose a significant microbiological complexity in the endodontic field. Silver nanoparticles (AgNPs) have emerged as a promising irrigant option in root canal treatments; however, few studies are focusing on endodontic biofilms. This work aimed to evaluate the antimicrobial and anti-adherence properties of AgNPs against clinically isolated bacteria taken directly from patients with various pulp and periapical diseases. **Methods:** AgNPs of two sizes were synthesized and characterized. The bactericidal and anti-adherence activities of AgNPs were evaluated through microbiological assays using experimental in vitro and ex vivo tests on oral biofilms taken from patients with symptomatic apical periodontitis (AAP) and pulp necrosis (PN). NaOCl solution was used as the gold standard. **Results:** The size of AgNPs was uniformly distributed (13.2 ± 0.4 and 62.6 ± 14.9 nm, respectively) with a spherical shape. Both types of nanoparticles exhibited good antimicrobial and anti-adherence activities in all microbiological assays, with a significant difference from NaOCl for in vitro and ex vivo models (*p* < 0.05). The inhibitory activity of AgNPs is mainly related to the type of microbiological sample and the exposure time. The antibacterial substantivity of both nanoparticle sizes was time-dependent. **Conclusions:** AgNPs may represent a promising antimicrobial option as an endodontic irrigant during conventional root canal treatments to prevent and control endodontic infections.

## 1. Introduction

Endodontic infections represent a serious health problem during the disinfection of conventional endodontic therapies, leading to considerable failures and poor long-term success of endodontic treatments [1]. Several endodontic alterations are considered a great challenge due to resistant microorganisms, having a microbiological organization distributed in a biofilm. A biofilm is a sessile multicellular microbial community characterized by cells that adhere firmly to the surface and are included in a matrix of extracellular polymeric substances called exopolysaccharides (EPS) [2]. These are structurally and dynamically organized biological systems. Biofilms can contain up to 300 layers and form microcolonies, which may consist of a single species but are generally mixed [2,3]. The extracellular matrix serves as structural support and is biologically active. This matrix helps retain nutrients, water, and essential enzymes within the biofilm, protects the bacterial community from external threats, and plays a role in surface adhesion [2,3,4]. It is estimated that 65% to 80% of human infections are associated with biofilms. Diseases such as gingivitis, dental caries, or periodontitis are linked to these bacterial communities in the oral cavity [5]. There is increasing evidence that apical periodontitis (AP) is also initiated by biofilms [6], while pulp necrosis (PN) precedes apical periodontitis [3], infecting the root canal and maturing the biofilm. Once they colonize the canals, they exit through the dental apex and initiate AP [3,6,7]. However, given the complexity of root canal anatomy, they can also grow in accessory and lateral canals, apical deltas, recurrent, collateral, and secondary canals, making access to them more challenging [8,9]. For this reason, root canal treatment aims to penetrate as many canals and dentinal tubules as possible using instruments, medications, irrigating agents, and irrigation techniques.

*Enterococcus faecalis* (*E. faecalis*) is one of the most frequently encountered Gram-positive bacteria implicated in recurrent and constant endodontic infections, and it is strongly associated with failures of conventional root canal treatments [2,10]. Moreover, *E. faecalis* is associated with a spectrum of life-threatening diseases such as infective endocarditis and urinary tract infections [11,12]. Authors have recommended a mechanism for oral biofilm development that includes *E. faecalis* bacteria [2,13]. The endodontic biofilm starts with planktonic bacteria adhering to the stable substrate, gradually aggregating and irreversibly incorporating more and different species of microorganisms, in which they form microcolonies using an EPS matrix as a robust anchoring structure. Subsequently, bacterial aggregation and strong adherence to the substrate improve a mature architecture with a high bacterial density. This results in a well-formed three-dimensional biofilm, biochemically regulated by quorum sensing (QS). In the final stage, some microcolonies are released from the initial biofilm, traveling freely to new surfaces and spreading the bacterial infection to other areas [2,13]. Particularly, the QS associated with *E. faecalis* plays a significant biochemical function in regulating the formation and survival of the endodontic biofilm [14]. This mechanism of bacterial clustering to develop the endodontic biofilm related to *E. faecalis* continues to pose a serious challenge for various antimicrobial agents in endodontics [3,7].

During conventional endodontic therapies, several chemical solutions are commonly used for disinfecting and irrigating root canals. Various authors have positioned the sodium hypochlorite (NaOCl) solution as the gold standard in endodontics, due to its excellent antimicrobial activity [8,15,16]. However, this solution has also shown deficiencies in removing the smear layer [17] and, in some cases, damaging the intratubular surface in the root canal and reducing the mechanical strength of dentin tissue, which increases the risk of root fracture in conventional root canal treatments [9]. Additionally, the extrusion of NaOCl solution into the periapical area is an endodontic accident with undesirable effects, leading to severe and localized pain with diffuse swelling and hemorrhage [18]. Consequently, new alternatives using novel antibacterial approaches focused on controlling endodontic infections need exploration. Nanomaterials have provided an alternative method to combat infections, offering advantages in dentistry [19,20]. Silver nanoparticles (AgNPs) are a nanostructured material widely studied due to their excellent antimicrobial properties [21,22], including planktonic and biofilm-related to oral species [17,23,24,25,26]. Available literature agrees with the outstanding antimicrobial and anti-biofilm benefits of AgNPs against oral microorganisms, indicating a potential antimicrobial alternative in the dental field [17,23,24,25,26,27]. Nonetheless, there is limited scientific information regarding the antimicrobial and anti-adherence properties of AgNPs against clinically isolated oral biofilms with endodontic and perio-endodontic alterations. The aim of this study was to determine the antibacterial and anti-adherence effects of AgNPs on clinical biofilms isolated from patients with endodontic and perio-endodontic alterations using in vitro and ex vivo models. These findings will contribute to a deeper understanding of the antimicrobial and anti-biofilm effects of AgNPs in dentistry, defining their safe use and predicting the mechanism of novel endodontic therapeutic tools for the disinfection processes during conventional root canal treatments.

## 2. Materials and Methods

### 2.1. Preparation and Characterization of AgNPs

AgNP synthesis was performed following a previously described method [24]. Two families of AgNPs were prepared using aqueous methods. The first set of nanoparticles was synthesized using 0.169 g (0.01 M) of silver nitrate (AgNO_3_, CTR Scientific, Monterrey, Mexico) dissolved in 100 mL of deionized water (DW) and stirred magnetically for 5 min. Then, 10 mL of deionized water containing 0.1 g (0.05 M) of gallic acid (C_7_H_6_O_5_, Sigma Aldrich, St. Louis, MI, USA), acting as both a reducing and stabilizing agent, was added to the mixture. The pH was adjusted to 11 using 1.0 M sodium hydroxide (NaOH, Jalmek Scientific, San Nicolás de los Garza, Mexico). For the second set, the gallic acid concentration was increased to 0.5 g (0.2 M). Ammonium hydroxide (NH_4_OH, molecular weight 35.05, catalog no. A5325, CAS no. 1336-21-6, Jalmek Scientific, San Nicolás de los Garza, Mexico) was used in place of sodium hydroxide to adjust the pH to 10.0. Both hydroxide solutions contributed to stabilizing the particle size in their respective systems. DW was employed in the synthesis of AgNPs to minimize the risk of unwanted chemical reactions, the formation of precipitates, and the contamination of the solution, while also helping to control the stability, morphology, and distribution of the nanoparticles. Particle size, size distribution, and electrical characteristics of each reaction mixture were analyzed via dynamic light scattering (DLS) using a Nanoparticle Analyzer (Nano Partica SZ-100 series, HORIBA Scientific Ltd., Irvine, CA, USA). UV-Vis absorption spectra of the nanoparticles suspended in water at room temperature were acquired with a Cary 100 spectrophotometer (Varian Corp., Cary, NC, USA) using a 10 mm quartz cuvette. In addition, particle morphology and elemental composition were examined using transmission electron microscopy (TEM, Philips CM-200, Philips Electronics NV, Eindhoven, The Netherlands) operated at an accelerating voltage of 25 kV, in conjunction with energy-dispersive X-ray spectroscopy (EDX).

### 2.2. Collection of Endodontic Biofilms

Oral biofilm samples were obtained from the Endodontic Clinics of the Stomatology Department at the Institute of Biomedical Sciences (ICB), Autonomous University of Ciudad Juárez (UACJ), Mexico. Each patient provided individual, written, and voluntary informed consent before sample collection, under the ethical principles outlined in the Declaration of Helsinki (2024). The Bioethics Committee of the ICB-UACJ revised and approved this study (project ID RIPI2022ICB10). The research included three oral biofilms from adult subjects (one from a male and two from female patients) aged between 40 and 55 years, who received dental care due to dental pain, endo-periodontal disease with pulp necrosis, periapical alteration, or previous root canal treatment. The oral biofilms included were one related to pulp necrosis (PN) and two samples for asymptomatic apical periodontitis (AAP). The *Enterococcus faecalis* microorganism (*E. faecalis*, ATCC 29212™, American Type Culture Collection, Manassas, VA, USA) was used as a reference strain. A specialist and expert in endodontics previously diagnosed the presence and types of endodontic and perio-endodontic biofilms.

The endodontic biofilms were obtained by inserting sterilized endodontic absorbent paper points (#20 Ref/UP 1560, Ultradent Products Inc., South Jordan, UT, USA) into each root canal of the teeth up to the apical zone for 10 s. Then, the type K endodontic files (#15, 25 mm, Maillefer, Dentsply, SWI) were used to remove softened dentin from root canals. Each patient’s paper points and endodontic files were immediately immersed in Mueller–Hinton broth (MH, BD™ Difco™, Rockville, MD, USA), cultured, and incubated for anaerobic conditions at 37 °C for 24 h.

### 2.3. Standardization of Bacterial Suspension

A standardized bacterial suspension of 1.5 × 10^8^ colony-forming units per milliliter (CFU/mL) was prepared using spectrophotometric measurement at 550 nm (Eppendorf BioPhotometer Plus, Hamburg, Germany). The suspension was then diluted to a final concentration of 1.5 × 10^6^ CFU/mL, which was used in all in vitro and ex vivo experiments.

### 2.4. Antimicrobial Test of AgNPs on Oral Biofilms

The antibacterial test was conducted following a previously published method [22]. All endodontic samples were incubated in Mueller–Hinton (MH) broth at 37 °C for 24 h before experimentation. Minimum biofilm inhibitory concentrations (MBICs) were determined by incubating bacterial samples treated with different antimicrobial agents in 96-well microdilution plates, each containing 100 µL of MH broth. Initially, 200 μL of each experimental AgNP solution was added to the first column of the microdilution plate, followed by serial dilutions up to the eleventh column. Subsequently, 100 μL of a standardized bacterial suspension (~1.5 × 10^6^ CFU/mL) was added to every well. The plates were then incubated at 37 °C for 24 h. The first column served as the negative control (no bacterial growth), while the twelfth column was the positive control (bacterial growth). MBIC values were determined by assessing turbidity through visual and stereoscopic examination of the last well exhibiting bacterial growth, compared against the control columns. A 5.25% sodium hypochlorite solution (NaOCl, Viarzoni-T, Viarden™, MX) was employed as the gold standard under the same assay conditions. All antimicrobial experiments were conducted in triplicate.

### 2.5. Antimicrobial Substantivity of AgNPs

The antimicrobial substantivity assay was performed using established parameters with some modifications [28]. A total of 20 μL of each antimicrobial sample (AgNP, NaOCl, and deionized water used as a negative control) and 100 μL of a standardized suspension (1.3 × 10^8^ CFU/mL) of endodontic biofilms were added to sterilized tubes containing 4 mL of MH broth. The tubes were incubated at 37 °C and examined by a UV-Vis spectrometer (spectrophotometer, Bibby Scientific™, Jenway™, 7305, Stone, Staffordshire, UK) to measure changes in optical density (OD) at 550 nm after 0, 0.5, 2, 7, 24, and 48 h. The final concentration for both AgNP and NaOCl samples was 5.1941 μg/mL. Substantivity was evaluated by monitoring changes in absorbance units (a.u.) reflecting the inhibition of endodontic biofilm growth in samples treated and untreated with AgNPs. All procedures were performed in triplicate.

### 2.6. Anti-Adherence Activity of AgNPs in an Ex Vivo Model

Twelve freshly extracted, single-rooted teeth were obtained through a non-probabilistic consecutive sampling method and preserved in 0.85% NaOCl saline solution to prevent dehydration. Teeth exhibiting visible structural alterations or existing restorations were excluded from the study. Subsequently, all samples were thoroughly cleaned, sonicated, and stored in saline solution at 4 °C. The preparation of samples started with removing anatomic crowns with carbide discs using irrigation. Then, all root canals from radicular portions were morphologically prepared with rotatory endodontic files (ProTaper Next, Dentsply Sirona, MI, USA) according to the manufacturer’s recommendations and were finally sterilized. Afterward, root canals were immersed in 20 mL of MH broth and infected with 200 µL of a standardized bacterial suspension (1.3 × 10^8^ CFU/mL) for each endodontic biofilm (PN, AAP, and a reference strain of *E. faecalis*). All root canals were refreshed with MH broth and reinfected with new endodontic bacteria from each endodontic biofilm using the standardized suspension described above, twice daily for two days. The root samples were incubated and agitated in a digital shaking incubator (Labnet 311DS, Labnet International, Edison, NY, USA) at 37 °C with 120 rpm.

Once the root canals were infected with specific endodontic biofilms, root samples were randomly assigned to the different antibacterial treatments. The antibacterial endodontic therapy included the irrigation of infected root canals with each antimicrobial solution, using a standard concentration (1070 µg/mL for AgNP, and 52,500 µg/mL for NaOCl) according to clinical endodontic recommendations [29]. The antimicrobial solution irrigation was made using an endodontic syringe (Monoject™, Covidien™, Mansfield, MA, USA) with a side outlet dental needle (Endo-Eze™, Ultradent™), applying 3 mL of each solution in six intervals of 500 µL. Deionized water was used as the control group under equivalent conditions. Following this, each root sample was placed in an individual tube containing 1 mL of PBS and subjected to sonication for 10 min. The resulting bacterial suspensions were diluted at a ratio of 1:100,000, and 100 µL of each dilution was plated on Mueller–Hinton agar (MH, BD™ Difco™, Rockville, MD, USA) and incubated at 37 °C for 24 h. The anti-adherence properties of AgNPs were evaluated by quantifying colony-forming units per milliliter, with all assays performed in triplicate.

### 2.7. Statistical Analysis

Data from age, DLS measurements, zeta potential, antimicrobial activity, substantivity, and anti-adherence effects were reported as means with standard deviations. The Shapiro–Wilk test was applied to assess the normality of values obtained from both in vitro and ex vivo experiments. Differences among antimicrobial groups were analyzed using the Mann–Whitney U test and the Kruskal–Wallis test, followed by appropriate post hoc comparisons. All statistical analyses were performed using IBM SPSS software (version 25, Chicago, IL, USA), with significance set at *p* < 0.05.

## 3. Results

### 3.1. Physical Characterization of AgNPs

**Figure 1:**
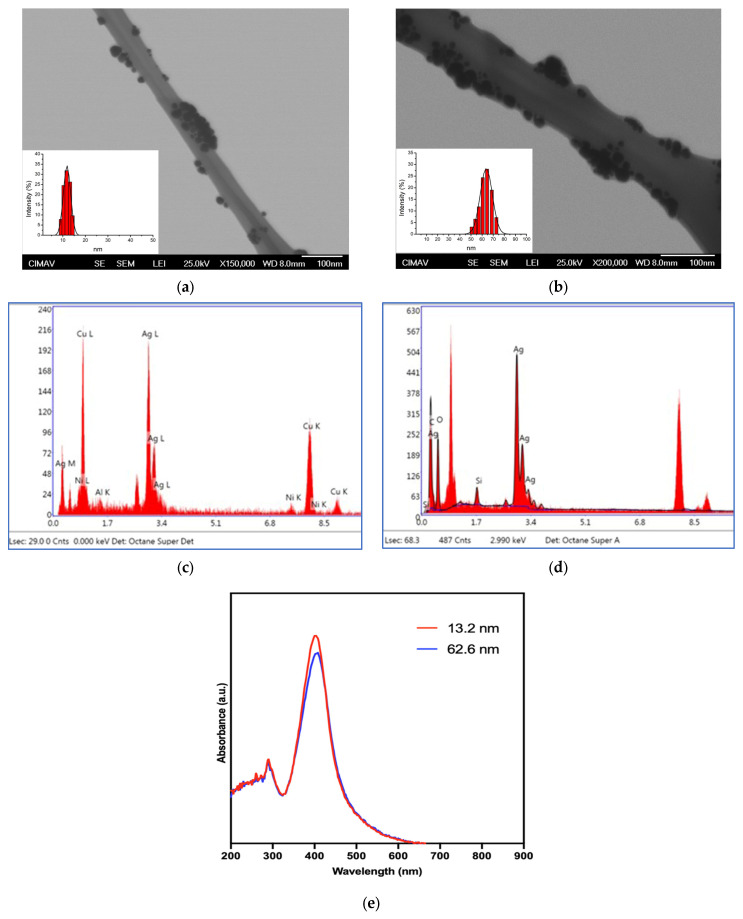
Characterization of AgNPs by TEM and DLS. (**a**) 13.2 nm, X30,000; (**b**) 62.6 nm, X40,000; (**c**) EDX for 13.2; and (**d**) EDX 62.6 nm. (**e**) UV-Vis analysis.

Table 1 and Figure 1 exhibit the physicochemical characteristics of AgNP. According to the DLS, two types of AgNPs (13.2 ± 0.4 and 62.6 ± 14.9 nm, respectively) with a narrow particle size distribution (Table 1), small bases, and a spherical shape were obtained (Figure 1a,b). Results from zeta potential indicate that both families of nanoparticles show negative values with acceptable and well-defined electrical charges (−62 ± 19.5 and −72.0 ± 3.9 mV) with a considerable presence of Ag in the elemental distribution according to EDX (43.46 and 31.86% in total weight, respectively) (Figure 1c,d). The UV-Vis spectra of both families of AgNPs display the characteristic surface plasmon resonance (SPR) typical for silver-based nanoparticles, with a maximum absorbance at 402 and 408 nm, respectively (Figure 1e). The bathochromic shift (towards a longer wavelength) of the SPR is due to the increase in the size of the nanoparticles. The presence of this surface plasmon resonance indicates that the AgNPs are well-dispersed and stable; nonetheless, the detection of additional absorption bands suggests that residual compounds may also be present.

### 3.2. Bactericidal Activity of AgNPs in Endodontic Biofilms

**Figure 2:**
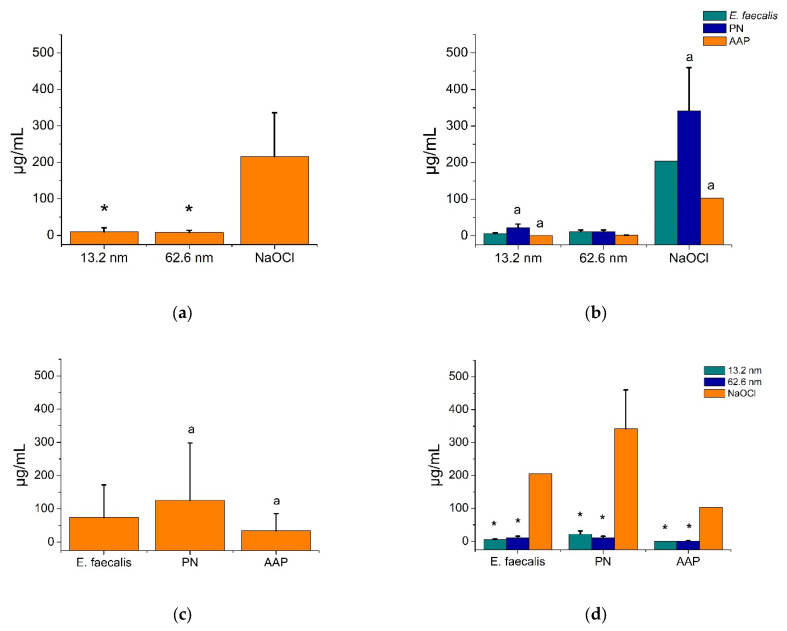
Bactericidal action of AgNPs and NaOCl in endodontic biofilms and *E. faecalis* strain (ATCC 29212). PN = pulp necrosis; AAP = asymptomatic apical periodontitis. Comparison between antimicrobial groups was determined by the Mann–Whitney U test. An asterisk denotes statistical differences with the control group (NaOCl). Similar letters show statistical differences between groups (*p* < 0.05). (**a**) General bactericidal activity of antimicrobial treatments. (**b**) Bactericidal activity of antimicrobial treatments according to endodontic biofilms. (**c**) Microbial resistance of endodontic biofilms. (**d**) Microbial resistance of endodontic biofilms according to antimicrobial treatments.

Figure 2 shows the MBIC results for AgNPs and NaOCl against endodontic biofilms and the *E. faecalis* strain. All antibacterial agents exhibited strong antimicrobial activities against all endodontic biofilms and the reference bacterial strain; however, both types of AgNPs demonstrated superior bacterial growth inhibition activities (7.9–9.6 ± 5.9–10.8 µg/mL, respectively) compared to NaOCl (216.3 ± 119.5 µg/mL) (Figure 2a), even for all bacterial strains (*p* < 0.05) (Figure 2d). Likewise, the PN biofilm showed higher levels of antimicrobial resistance (125.0 ± 173.0 µg/mL) compared to the *E. faecalis* strain (73.9 ± 98.3 µg/mL) and the AAP biofilm (34.9 ± 50.6 µg/mL). Consequently, the PN sample was statistically the most resistant biofilm, while the AAP sample was the most sensitive to all antimicrobial agents (Figure 2c,d) (*p* < 0.05). Particularly in the larger AgNP (62.6 nm), all biofilms and the reference bacterial strain had similar levels of antimicrobial resistance (*p* > 0.05) (Figure 2b). These findings indicate that AgNPs had better antimicrobial activity than NaOCl for all endodontic biofilms and the reference strain. The PN biofilm was the most resistant endodontic biofilm for all antimicrobials, and the AAP was the most sensitive, highlighting the relationship between the type of antimicrobial agent and type of bacterial strain as key factors influencing the bactericidal action of both AgNPs and NaOCl solutions.

### 3.3. Antimicrobial Substantivity of AgNPs in Endodontic Biofilms

**Figure 3:**
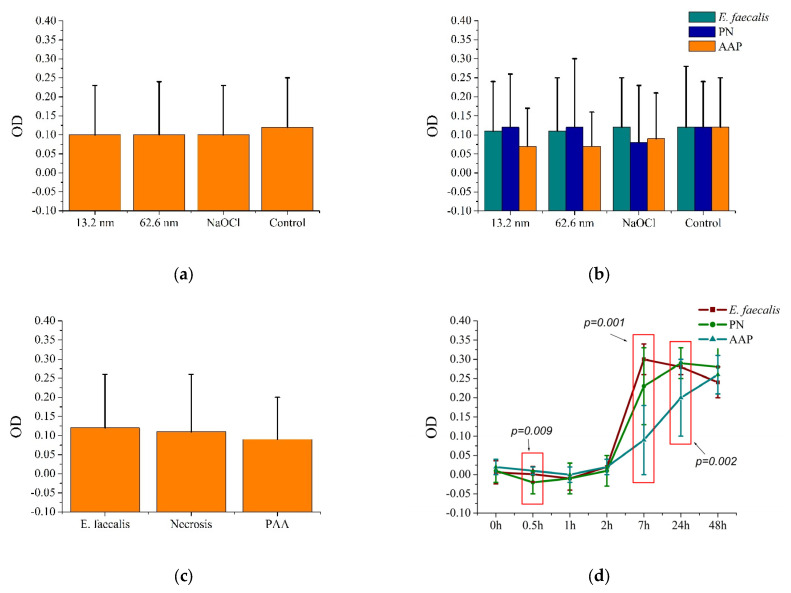
Antimicrobial substantivity of AgNPs against endodontic biofilms and the reference strain of *E. faecalis* (ATCC 29212). PN = pulp necrosis; AAP = asymptomatic apical periodontitis. All values are measured in absorbance units (a.u.) from optical density (OD) and expressed as means with standard deviations. For independent and grouped comparisons, the Mann–Whitney U and the Kruskal–Wallis tests were applied (*p* < 0.05). (**a**) Antimicrobial substantivity of antimicrobial treatments. (**b**) Antimicrobial substantivity of antimicrobial treatments according to endodontic biofilms. (**c**) Microbial resistance of endodontic biofilms. (**d**) Microbial resistance of endodontic biofilms according to time.

Figure 3 explains the growth inhibition substantivity of AgNPs and NaOCl in endodontic biofilms and *E. faecalis*. As noted, all antimicrobial agents had similar bacterial growth rates for all bacterial strains (0.10 ± 0.13–0.14 a.u.), while the control group (deionized water) showed the lowest antimicrobial efficacy (0.12 ± 0.13 a.u.), but no statistical significance was observed (*p* > 0.05) (Figure 3a). Meanwhile, *E. faecalis* was the most resistant strain (0.12 ± 0.14 a.u.), followed by PN (0.11 ± 0.15 a.u.), with the AAP strain as the most sensitive endodontic biofilm (0.09 ± 0.11 a.u.). Although there were varying levels of antimicrobial resistance or sensitivity to the antimicrobials, no statistical differences were described (*p* > 0.05) (Figure 3c). Both sizes of AgNPs had similar antimicrobial responses among all bacterial strains; however, NaOCl solutions tended to display more antimicrobial efficacy (0.08 ± 0.15 a.u.) than AgNPs against the PN biofilm (0.11 ± 0.13 and 0.11 ± 0.14 a.u., respectively), although no significant associations were observed (*p* > 0.05) (Figure 3b). Furthermore, the bacterial growth rates for all bacterial strains exposed to antimicrobial agents were observed (Figure 3d). At 0.5 h, the microorganisms within the PN biofilm had a statistically lower bacterial proliferation rate (−0.02 ± 0.03 a.u.) compared to the AAP (0.01 ± 0.01 a.u.) biofilm and *E. faecalis* (0.0012 ± 0.02 a.u.); however, at 7 and 24 h, *E. faecalis* (7 h = 0.3 ± 0.04 and 24 h = 0.28 ± 0.02 a.u.) and the PN biofilm (7 h = 0.23 ± 0.1 and 24 h = 0.29 ± 0.04 a.u.) presented significantly more antimicrobial resistance than the AAP biofilm (7 h = 0.09 ± 0.09 and 24 h = 0.2 ± 0.1 a.u.) (Figure 3d). These results suggest that while there are variations according to specific bacterial samples, exposure time, particularly at 7 and 24 h, statistically determines the sensitivity or resistance levels of specific bacteria or biofilms when exposed to the antimicrobial agents.

**Figure 4:**
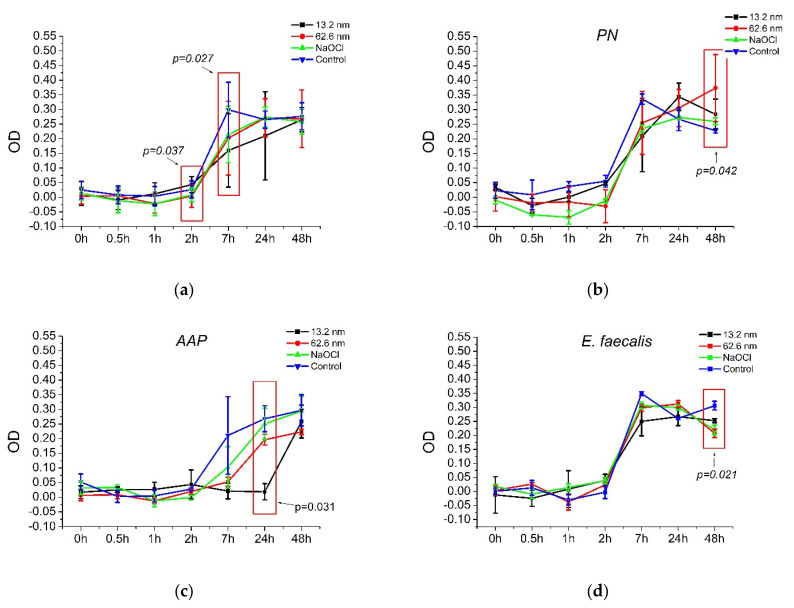
Bactericidal substantivity of AgNPs in endodontic biofilms, including a reference strain of *E. faecalis* (ATCC 29212). PN = pulp necrosis; AAP = asymptomatic apical periodontitis. Statistical differences between groups were evaluated using the Kruskal–Wallis test (*p* < 0.05). (**a**) General antimicrobial substantivity. (**b**) Antimicrobial substantivity in PN biofilm. (**c**) Antimicrobial substantivity in AAP biofilm. (**d**) Antimicrobial substantivity in *E. faecalis* biofilm.

Table 2 and Figure 4 describe the bactericidal substantivity of AgNPs and NaOCl against endodontic biofilms and a reference strain of *E. faecalis*. As observed, all antimicrobial agents showed statistically similar antimicrobial activities for all bacterial samples (*p* = 0.292–0.939), while the endodontic biofilms, including the reference strain of *E. faecalis*, presented no significant differences according to the resistance levels to each antimicrobial solution (*p* = 0.141–0.689) (Table 2).

According to specific times, at 2 h, the larger AgNPs (0.004 ± 0.03 a.u.) and NaOCl solution (0.008 ± 0.02 a.u.) exhibited higher growth rates than the smaller AgNPs (0.04 ± 0.02 a.u.) and the control group (0.02 ± 0.02 a.u.). However, at 24 h, the most effective antimicrobial agent was the smaller AgNPs (0.209 ± 0.15 µg/mL), followed by the larger AgNPs (0.271 ± 0.06 a.u.), NaOCl (0.274 ± 0.03 a.u.), and, finally, the control group (0.265 ± 0.02 a.u.) (Figure 4a). Particularly in the PN biofilm, significant differences were observed at 48 h, showing more bacterial growth in large nanoparticles (0.374 ± 0.11 a.u.) than the other antimicrobial agents (0.227–0.284 ± 0.007–0.05 a.u.) (Figure 4b). In the AAP biofilm, statistical differences were found at 24 h, in which the smaller nanoparticles exhibited more antimicrobial substantivity (0.018 ± 0.027 a.u.) compared to the larger ones (0.196 ± 0.018 a.u.), NaOCl (0.250 ± 0.053 a.u.), and the control group (0.268 ± 0.044 a.u.), respectively (Figure 4c). Finally, for the reference strain of *E. faecalis*, significant differences were observed also at 48 h, identifying that all antimicrobial agents showed statistically similar antimicrobial substantivity (small AgNP = 0.252 ± 0.007 a.u., large AgNP = 0.207 ± 0.015 a.u., and NaOCl = 0.219 ± 0.017 a.u.), although with an improved bacterial growth inhibition relative to the control group (0.306 ± 0.015 a.u.) (Figure 3d). These findings suggest that the antimicrobial substantivity of bactericidal solutions might directly depend on the type of endodontic biofilm and bacteria, the bactericidal agent, and the time of exposure to the antimicrobials.

### 3.4. Anti-Adherence Activity of AgNPs in an Ex Vivo Model

**Figure 5:**
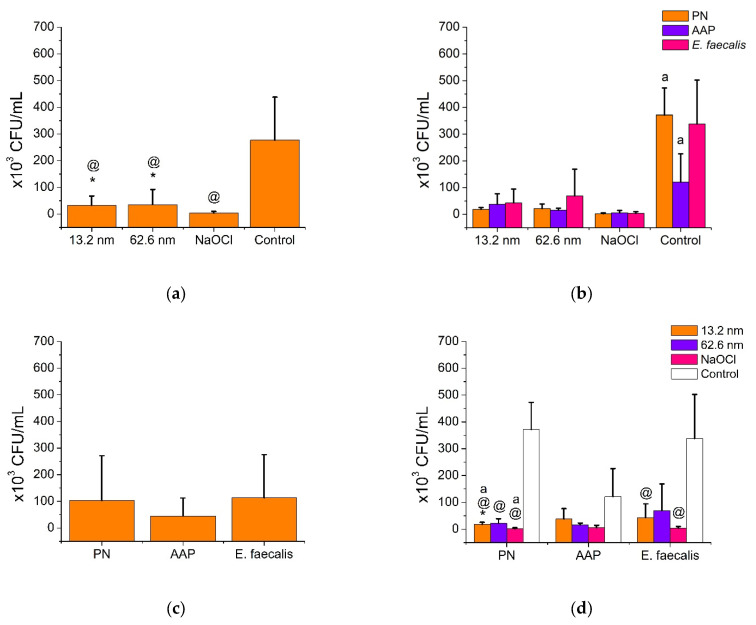
Anti-adherence activity of AgNPs against endodontic biofilms and *E. faecalis* (ATCC 29212). PN = pulp necrosis; AAP = asymptomatic apical periodontitis. The Mann–Whitney U test was employed for pairwise comparisons between independent groups. An asterisk (*) denotes statistically significant differences relative to the positive control (NaOCl), while the symbol (@) indicates significant differences compared to the negative control (deionized water). Groups sharing the same letter are significantly different from one another (*p* < 0.05). (**a**) Anti-adherence activity of antimicrobial treatments. (**b**) Anti-adherence activity of antimicrobial treatments according to endodontic biofilms. (**c**) Anti-adherence resistance of endodontic biofilms. (**d**) Anti-adherence resistance of endodontic biofilms according to antimicrobial treatments.

**Figure 6:**
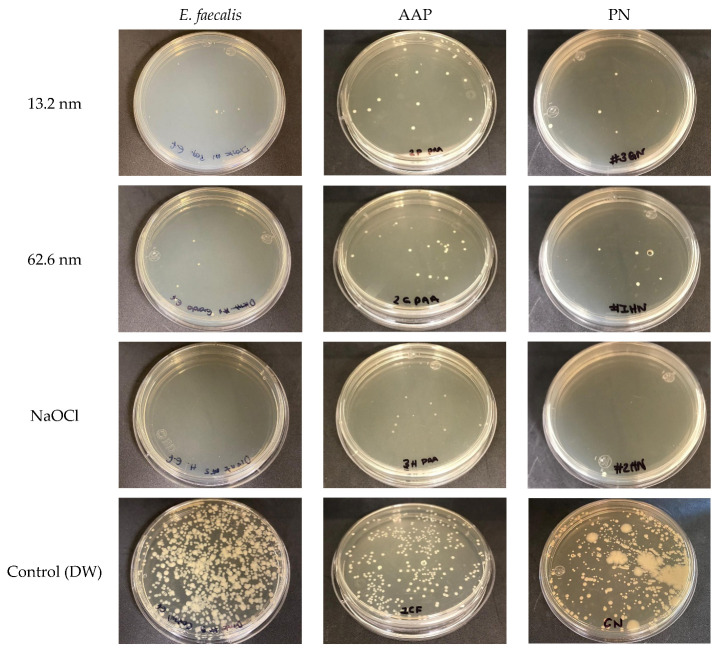
Representative images of anti-adherence action of AgNPs in endodontic biofilms and a reference strain of *E. faecalis* (ATCC 29212). PN = pulp necrosis; AAP = asymptomatic apical periodontitis.

Figure 5 and Figure 6 show the anti-adherence activity of AgNPs in endodontic biofilms and a reference strain of *E. faecalis*. As observed, all antimicrobial solutions exhibited effective and comparable anti-adherent activities against each endodontic biofilm and *E. faecalis* (*p* > 0.05), while the PN biofilm and the *E. faecalis* showed the highest levels of adherence (Figure 5). Particularly, all antimicrobial agents showed superior anti-adherence activity (AgNP 13.2 nm = 33.2 ± 34.2 CFU/mL, AgNP 62.6 nm = 35.5 ± 56.6 CFU/mL, and NaOCl = 3.6 ± 6.0 CFU/mL) compared to the deionized water (276.8 ± 161.1 CFU/mL) (*p* < 0.05), with the NaOCl solution as the most effective anti-adherent agent, followed by both types of AgNPs (Figure 5a). In addition, each antibacterial solution had comparable anti-adherent effects for all endodontic biofilms and the reference strain of *E. faecalis* (*p* > 0.05). Therefore, in the deionized water group (control group), the PN biofilm (371.6 ± 101.2 CFU/mL) and the reference strain of *E. faecalis* (338.0 ± 164.6 CFU/mL) showed higher adherent activities than the AAP biofilm (121.0 ± 105.0 CFU/mL) (Figure 5b,d). Adherence variations between bacterial samples were also found, with the reference strain of *E. faecalis* showing a higher level of adhesion (113.6 ± 161.5 CFU/mL), followed by the PN biofilm (103.4 ± 167.8 CFU/mL) and finally, the AAP biofilm, with the lowest adhesion (44.9 ± 67.5 CFU/mL). However, no significant differences in the levels of bacterial adhesion were established (Figure 5c). Representative agar plates show good anti-adherent activity of both sizes of AgNPs in comparison with NaOCl solution and the control group, indicating an effective control of bacterial proliferation and low intracanal adhesion (Figure 6). These findings suggest that the anti-adherent activity of AgNPs is influenced by the type of endodontic bacterial sample (biofilm or reference strain) and the type of antimicrobial solution used, determining the variations in anti-adherent properties based on specific conditions between the antimicrobial endodontic therapy and the specific endodontic bacteria related to the endodontic diagnosis.

## 4. Discussion

This work confirmed that both families of AgNPs effectively suppressed the growth of microorganisms found in oral biofilms collected from patients with different endodontic conditions. Additionally, the best antimicrobial effect of AgNPs was associated with small particle size in specific cases only. Thus, oral biofilms from patients with PN harbored the most resistant bacteria to all antimicrobial treatments, including AgNPs, while those from individuals with AAP contained the most susceptible bacterial populations. On the other hand, some differences in bacterial growth rate were found depending on specific bacterial samples. The exposure time, especially after 7 and 24 h, determines roles in defining the sensitivity or resistance levels of certain bacteria or biofilms to antimicrobial agents. In addition, the anti-adherent activity of AgNPs determined that silver solutions can decrease bacterial adherence in both endodontic biofilms, even for *E. faecalis*, compared to NaOCl solution. Interestingly, the endodontic reference strain of *E. faecalis* and the PN biofilm presented the highest adherence resistance, while the AAP biofilm showed the lowest adherence levels. To our knowledge, this is the first investigation to assess the antimicrobial and anti-adherence properties of AgNPs with two distinct particle sizes against diverse oral biofilms derived from patients with endodontic and periapical pathologies. These findings promote an improved estimation of the bactericidal and anti-adherence mechanisms of AgNPs when interacting with microorganisms within endodontic biofilms under clinically relevant microbiological conditions. Furthermore, the ability of AgNPs to inhibit bacterial growth, combined with their physicochemical interactions with oral clinical biofilms, may support the prevention and management of endodontic infections, ultimately contributing to improved oral health outcomes.

Physical and chemical properties represent relevant conditions for adequate activity against microorganisms. The prepared AgNPs in two different particle sizes were appropriate, as well-defined peaks with narrow bases were obtained. The zeta potential of both nanoparticles showed negative values with acceptable electrical charges to prevent nanoparticle aggregation. It is established that AgNPs possessing a surface charge between +31 and −30 mV, along with a high pH value above 7, facilitate the dispersion of colloidal silver and help prevent particle aggregation [30]. Also, gallic acid plays an important role as a reducing agent of silver ions, forming and covering the surface of nanoparticles [31,32]. Some authors have reported that gallic acid has been widely explored for several biomedical applications, including antioxidant, anticarcinogenic, antimutagenic, lipophilic, antimicrobial, and pro-oxidative properties [33,34], acting synergistically with other compounds, including nanoparticles [35]. In this study, highly negative surface charges were obtained (−62.5 and −72 mV), which could be due to factors such as the reducing agent (gallic acid) and the high pH levels (10 and 11). It is well known that both factors can provide functional groups on the particle surface that tend to ionize, generating negative charges with relatively high electric intensities [30,31,36]. Negatively charged particles may interact more effectively with bacteria due to their good dispersion and stability in solution, as indicated by high-intensity zeta potentials, resulting in larger surface areas without particle agglomeration [30,31]. Although bacterial membranes also carry negative charges, these particles can infiltrate biofilms or extracellular spaces before making contact with bacterial membranes [37]. At the same time, small particle size, aqueous media, the presence of oxygen, and other organic compounds such as proteins and biomolecules facilitate alternative mechanisms such as the oxidation of the nanoparticles, leading to the release of Ag+ ions [37]. These ions cause oxidative damage through the generation of reactive oxygen species (ROS), disrupt enzymes, DNA, and membrane structures, and consequently result in bacterial cell death [37,38].

Previous reports have widely defined that the AgNP’s bactericidal activity is related to its size [39,40,41]; however, few works have evaluated the growth inhibition activity of AgNPs in dental biofilms used as a disinfection agent for endodontic therapy. Researchers have explored the antimicrobial efficacy of AgNPs when used as a final irrigant during root canal therapy on dental roots infected with *E. faecalis* [42]. These authors determined that AgNPs exhibit effective antimicrobial properties and can be used as irrigating agents in endodontics, reducing bacterial biofilm [42]. In other work, the antimicrobial effect of AgNPs was evaluated, analyzing a significant number of dental biofilms sampled from subjects with caries and periodontal conditions, suggesting that AgNPs demonstrated effective bacterial growth inhibition, in which the antimicrobial mechanism may be influenced by particle size, exposure time, and gender [28]. Another study evaluated the antimicrobial activity of AgNPs as an endodontic solution and as a medication against *E. faecalis* biofilm [17]. The 0.01% AgNP solution was found to have no detrimental effect on the biofilm membrane, showing comparable results to those observed in the saline solution group; however, no statistically significant differences were observed. This suggests that AgNPs may be more effective as an intracanal medication rather than as an endodontic irrigant [17]. Recently, the antimicrobial effect of AgNPs against biofilms presented in dental plaque from patients with periodontal disease, and collected from 60 patients, was reported, demonstrating acceptable antimicrobial activity in all samples and stating that the main significant associations in the antimicrobial efficacy were related to particle size and the type of oral biofilm [24]. Additional studies have indicated that 2% CHX combined with AgNPs for up to seven days results in a significant reduction in multispecies biofilms, including *E. faecalis*, *Streptococcus mutans*, *Lactobacillus acidophilus*, and *Actinomyces naeslundii* [43]. Moreover, some researchers have estimated the bactericidal and anti-adhesion activities of a poly(vinyl alcohol)-covered AgNP (AgNP-PVA) and farnesol, revealing that AgNPs with PVA exhibit bactericidal and anti-adherence properties against *E. faecalis*, *Candida albicans*, and *Pseudomonas aeruginosa* and may be an alternative as a secondary method for intracanal disinfection or to prevent biofilm establishment [26]. Our results indicate that both sizes of AgNPs possess statistically better growth inhibition activity (smaller 9.6 ± 10.8 µg/mL and larger 7.9 ± 5.9 µg/mL) compared to NaOCl solution (216.3 ± 119.5 µg/mL) for all endodontic biofilms. Furthermore, this study also determined statistically similar anti-adherence activities of AgNPs (smaller 33.2 ± 34.2 CFU/mL and larger 35.5 ± 56.6 CFU/mL) in an ex vivo model compared to NaOCl solutions (3.6 ± 6.0 CFU/mL) (*p* > 0.05). Our findings indicate that AgNPs possess antimicrobial and anti-adherence activities, with optimal effectiveness influenced by exposure duration, the specific type of endodontic biofilm, and, in some specific cases, particle size.

Although several studies have described the antibacterial action of AgNPs [39,44,45], the exact mechanism remains unclear. The antimicrobial effect of AgNPs is believed to result from the release of silver ions that attach to the bacterial cell wall and cytoplasmic membrane, enhancing permeability and ultimately leading to the disruption of the bacterial envelope [44,45,46]. The ability of AgNPs with gallic acid to synergistically attach to cell membranes is likely influenced by electrostatic forces and interactions with the bacterial adhesion system, such as EPS, enabling strong bonds with the carboxyl, phosphate, amino, hydroxyl, and thiol groups present in the cell membranes of bacterial cells [47]. Once silver ions penetrate the cell, they induce the production of reactive oxygen species, which interfere with ATP synthesis and cause cellular damage as well as DNA alterations [45,48]. Additionally, these ions can attach to membrane invaginations and, upon accumulation, induce cellular denaturation and organelle disruption, ultimately leading to cell lysis [5,46,49]. The ions may also interfere with bacterial signal transduction, triggering apoptosis and preventing bacterial multiplication [45,47]. Also, the catalytic activity of AgNPs in the formation of disulfide bonds in the reaction of oxygen molecules has been reported, in which the function of important proteins and enzymes is affected [46]. A study evaluated the phenotypic and transcriptional effects of AgNPs in *Pseudomonas aeruginosa* biofilm to determine ROS and EPS productions, via extracellular DNA (eDNA). These authors concluded that the use of high concentrations of AgNPs affected the EPS production associated with a low quantification of eDNA, producing disruption in the formation of biofilm [50]. In our study, microorganisms from the PN sample were the most resistant to all treatments, whereas bacteria from the AAP biofilms were the most sensitive (Figure 2). This could be attributed to the type of bacterial species associated with each disease and the area where the root canal was sampled [15]. The sample was taken directly from the root canal. As is known, in AAP, the infection is not confined to the canal but has extended beyond the apical foramen. This suggests that the most resistant bacteria may not have been included in the sample. In contrast, for the PN, the bacterial sample was collected from the most heavily infected area. Bacteria present in pulp necrosis are primarily strict anaerobes, with some facultative anaerobes, and rarely aerobes. A study analyzing 62 infected human root canals reported that nearly all root canal infections are polymicrobial, with predominant bacterial species including *Porphyromonas gingivalis*, *Porphyromonas endodontalis*, and *Prevotella buccae* [15]. In root canals already affected by apical periodontitis, the predominant bacteria are strict anaerobes such as *Porphyromonas asaccharolytica*, *Fusobacterium nucleatum*, *Eubacterium lentum*, and *Peptostreptococcus micros* [6]. It is important to highlight that AgNPs require a lower concentration than NaOCl to eliminate the bacteria used in this study (Figure 2). The bacterial growth kinetics test was conducted to evaluate bacterial proliferation, and the results showed significant bacterial growth at hour 7 (Figure 3 and Figure 4). However, the smaller and larger AgNPs maintained lower bacterial counts than the other treatments and the control group (Figure 2). This may be due to the antimicrobial action of AgNPs, as the smaller size has a high surface area, facilitating direct contact and the internalization mechanism to penetrate bacterial pores and membranes more easily, enabling membrane infiltration and causing cell lysis [45]. Some authors reported that the antibacterial activity of AgNPs is closely linked to the specific surface area of the particles; smaller particles demonstrate greater effectiveness than larger ones due to their higher surface area, which enhances contact with bacterial cells [51]. In addition, AAP was the most sensitive sample to the antimicrobial treatments (Figure 2c), which could be attributed to the bacterial species present. In contrast, PN and *E. faecalis* exhibited greater resistance. It is well known that *E. faecalis* is a highly resistant bacterium, and in this test, its resistance form was similar to the PN biofilm [17,42,52]. Bacterial growth kinetics revealed differences in proliferation depending on the biofilm type present. This trend could lead to the development of selective irrigation treatments, making root canal treatments more predictable. We assume that AgNPs can, synergistically with the gallic acid, develop both antibacterial and anti-adherence activities on the oral biofilms, impairing the growth and aggregation of endodontic bacteria in a bio-community, influenced particularly by smaller size, spherical shape, and silver ion release, with specific particle concentrations and differences among types of endodontic biofilm [23,26,53]. In particular, AgNPs combined with gallic acid may interact with bacterial membranes through electrostatic forces and EPS, via eDNA [50] and lipoproteins [54], binding to functional groups including carboxyl, phosphate, amino, hydroxyl, and thiol groups, penetrating cells, and generating reactive oxygen species (ROS) that disrupt ATP production, damage DNA, parallelly interfering with signal transduction, trigger apoptosis, and affect the ATP levels and protein denaturation, facilitating cell lysis and the alteration of biofilm [53].

In this research, the microbial adhesion assay was performed on extracted dental specimens (ex vivo test), allowing for an experimental study of bacterial behavior under antibacterial treatments in clinical conditions that closely resemble real-life scenarios. The anti-adherence results demonstrated a tendency for lower CFU counts in smaller AgNPs compared to larger ones (Figure 5 and Figure 6). In addition, the AgNP solutions needed lower concentrations (1070 µg/mL) to generate statistically better antimicrobial and anti-adherence effects than NaOCl, which habitually applies higher concentrations in the endodontic area (52,500 µg/mL). These findings agreed with previous studies that have reported that smaller AgNPs display [15] a superior antimicrobial efficacy [36,42,55,56]. In addition, it was also observed that the antimicrobial mechanism of AgNPs depended on the type of biofilm; however, for NaOCl, the antimicrobial behavior of the three endodontic biofilms was equivalent (*p* < 0.05). The bacterial anti-adhesion activity of the treatments varied across the biofilms. Particularly, the 13.2 nm AgNP exhibited improved results against PN and *E. faecalis* than the nanoparticles of 62.6 nm (*p* < 0.05). Interestingly, for AAP, the 62.6 nm nanoparticles were more efficient than the 13.2 nm ones. In addition, the bacteria in the AAP biofilm showed lower CFU formation (Figure 5 and Figure 6), which could be due to the method used to obtain the sample. This is likely because the internal bacterial distribution within the root canal differs from that in periapical areas of the pulpal condition. This suggests that apical periodontitis is specific to the periapex, originating in the root canal and progressing toward the apical tissues, forming a biofilm in the external periradicular portion of the root [3,6]. In this study, the sample was taken from the root canal without the periapical area. Due to this, it is suggested that other examinations must be performed using clinical samples taken from the root canal and specifically obtained from the root apex of teeth with apical periodontitis. This approach would enhance bacterial representation within the biofilm samples, enabling the identification of bacterial species present in the various regions of the root canal.

The AgNP’s cytotoxicity is one of the most important factors involved in the safe use in humans. It is known that these metallic nanoparticles trigger an immune response. When they are detected by lymphocytes and macrophages, proinflammatory cytokines such as IL-1, IL-6, and TNF are released, producing reactive oxygen species (ROS) and creating disruptions in the biological system [57,58]. Also, the presence of lipoproteins such as low and high density lipoproteins (LDL and HDL, respectively) with AgNPs produces modifications in their physicochemical and immunological responses, affecting biological functions [54]. The biodistribution and toxicity of AgNPs are the most important effects related to lipoproteins due to the rapid associations with plasmatic proteins in the body, modifying the dissolution of particles and consequently their toxicity [54,59]. Studies have indicated that the potential toxicity of AgNPs is influenced by multiple factors that contribute to oxidative stress and cytotoxicity, including particle size, concentration, exposure duration, the route of administration, dosage, particle type, and morphology, among others [60,61,62]. In addition, studies have determined the potential toxicity of AgNPs and ionic silver using human cells, including gingival and dermal fibroblast cells [63,64]. These authors conclude that ionic silver was more toxic in human cells than AgNPs, and a low concentration of AgNPs, such as 25 µg/mL, is more suitable for wound healing in dermatologic applications [63], but also, AgNPs are safe to healthy gingival fibroblast cells at a concentration <260 µg/mL [64]. In contrast, studies have reported that NaOCl can cause more oxidative DNA damage, producing more severe complications in patients at low concentrations [65,66]. NaOCl solutions have even demonstrated greater cytotoxicity levels than AgNPs [67,68]. Undoubtedly, other experiments should be conducted to define clearly the safe use of AgNPs in clinical conditions.

Other experimental approaches to AgNPs should be explored, in which long-term stability in physical, chemical, and microbiological properties must be studied. It is very well known that the stability of AgNPs can be affected by pH, ionic strength, temperature, ultraviolet light, and diverse organic compounds, among others [69]. Studies have reported that optimized conditions during storage, such as being stored in the dark and at room temperature, play a fundamental role in the long-term stability of AgNPs with good particle distribution and great antimicrobial effects after 2 and 6 months of storage [70]. In this study, AgNPs were stored for up to 3 months under dark conditions and room temperature using chemical stabilization with gallic acid according to recommendations from previous studies [70,71]. Despite this, both families of AgNPs could have good antimicrobial and anti-adherence stabilities over a long-term period, and, surely, some physicochemical and microbiological variations during clinical applications could appear [69]. Although the results of this study could suggest the use of AgNPs in the endodontic area as intracanal pastes, irrigant solutions, or incorporated in endodontic sealers, factors such as low representative biofilm sampling, types of endodontic diseases, a reference strain of *E*. *faecalis* (ATCC 29212), and sociodemographic and clinical aspects from biofilms, as well as in vitro and ex vivo models using AgNPs with particular physicochemical characteristics may act as limitations in the present study. Of course, more evaluations involving microbiological and toxicological assays, and more particle sizes, presentations, or therapeutics of AgNPs, as well as different endodontic biofilms from other endodontic infections with more representative sample sizes, using other in vitro, ex vivo, and in vivo models with more realistic simulated conditions or, even, complex scenarios (mammalian infection models or clinical studies), are needed. Even studies focused on machine learning-based identification in microbiomes or antimicrobial peptides have emerged as novel tools for the classification, prediction, pattern recognition, and control of infections related to biofilms [72,73]. These new methodological approaches will provide a better understanding of the safe use of AgNPs for clinical applications in endodontics.

## 5. Conclusions

AgNPs exhibited strong antibacterial activity, including growth inhibition and anti-adherence activity, against all oral biofilms associated with endodontic conditions. The antimicrobial effectiveness of AgNPs was, in some cases, induced by nanoparticle size and the type of endodontic biofilm. Statistical findings confirm that smaller and larger particles were the most efficient. Oral biofilms related to PN demonstrated the highest resistance to all antimicrobial treatments, while AAP biofilms were the most susceptible, particularly to the smaller nanoparticles. The antimicrobial substantivity of bactericidal solutions appears to depend on multiple factors, including the type of endodontic biofilm and bacterial configuration, the specific antimicrobial solution used, and the time of exposure to antimicrobials, with the highest activity for AgNPs observed after 7 h of exposure. Both types of AgNP displayed significant anti-adherence properties, which were linked to the endodontic biofilm and the antimicrobial solution used. AgNPs exhibited effective antimicrobial activity against PN and AAP endodontic biofilms and the *E. faecalis* strain, and their potential, as an alternative irrigant for controlling and preventing common and resistant endodontic infections, remains promising. When used alongside conventional root canal therapies, AgNPs could enhance treatment prognosis and long-term success in endodontics. Therefore, further innovative research on AgNP applications should be strongly encouraged.

## Figures and Tables

**Table 1:** Characterization of the AgNP families determined by DLS and TEM.

AgNP (nm)	Diameter DLS (nm)	Shape	Initial Concentration (μg/mL)	Zeta Potential (mV)
13.2	13.2 ± 0.4	Spherical	1070	−62.5 ± 19.5
62.6	62.6 ± 14.9	Spherical	1070	−72.0 ± 3.9

Values from DLS are expressed as the mean with standard deviation. DLS and TEM stand for dynamic light scattering and transmission electron microscopy, respectively.

**Table 2:** Antimicrobial substantivity of AgNPs against endodontic biofilms and a reference strain of *E. faecalis* (ATCC 29212).

Biofilm Strains	AgNP 13.2 nm	AgNP 62.6 nm	NaOCl (Ctrl+)	Deionized Water (Ctrl-)	*p*-Value
PN	0.12 ± 0.12	0.12 ± 0.18	0.08 ± 0.15	0.12 ± 0.12	0.292
AAP	0.07 ± 0.1	0.07 ± 0.09	0.09 ± 0.12	0.12 ± 0.13	0.361
*E. faecalis*	0.11 ± 0.13	0.11 ± 0.14	0.12 ± 0.13	0.12 ± 0.16	0.939
Total	0.10 ± 0.13	0.10 ± 0.14	0.10 ± 0.13	0.12 ± 0.13	0.406
*p*-Value	0.376	0.689	0.141	0.678	-

Optical density (OD) values were recorded in absorbance units (a.u.) and reported as mean data with standard deviations. Ctrl+ = positive control; Ctrl- = negative control; PN = pulp necrosis; and AAP = asymptomatic apical periodontitis. Statistical differences between groups were evaluated using the Kruskal–Wallis test (*p* < 0.05). - Not applicable.

## Data Availability

The raw data supporting the conclusions of this article will be made available by the authors on request. All data generated in this study are archived within the research records of the Master’s Program in Dental Sciences at the Autonomous University of Ciudad Juarez and are available upon request from the corresponding author.
